# Variations in Antioxidant Capacity, Oxidative Stability, and Physicochemical Quality Parameters of Walnut (*Juglans regia*) Oil with Roasting and Accelerated Storage Conditions

**DOI:** 10.3390/molecules27227693

**Published:** 2022-11-09

**Authors:** Youssef Elouafy, Zineb Lakhlifi El Idrissi, Adil El Yadini, Hicham Harhar, Mohammed Merae Alshahrani, Ahmed Abdullah AL Awadh, Khang Wen Goh, Long Chiau Ming, Abdelhakim Bouyahya, Mohamed Tabyaoui

**Affiliations:** 1 Laboratory of Materials, Nanotechnology and Environment LMNE, Faculty of Sciences, Mohammed V University in Rabat, Rabat BP 1014, Morocco; 2 Department of Clinical Laboratory Sciences, Faculty of Applied Medical Sciences, Najran University, P.O. Box 1988, Najran 61441, Saudi Arabia; 3 Faculty of Data Science and Information Technology, INTI International University, Nilai 71800, Malaysia; 4 PAP Rashidah Sa’adatul Bolkiah Institute of Health Sciences, Universiti Brunei Darussalam, Gadong BE1410, Brunei; 5 Laboratory of Human Pathologies Biology, Faculty of Sciences, Mohammed V University in Rabat, Rabat BP 1014, Morocco

**Keywords:** *Juglans regia*, walnut oil, oxidative stability, roasting, antioxidant activity, DPPH, tocopherol, phytosterol

## Abstract

Walnut oil, like all vegetable oils, is chemically unstable because of the sensitivity of its unsaturated fatty acids to the oxidation phenomenon. This phenomenon is based on a succession of chemical reactions, under the influence of temperature or storage conditions, that always lead to a considerable change in the quality of the oil by promoting the oxidation of unsaturated fatty acids through the degradation of their C–C double bonds, leading to the formation of secondary oxidation products that reduce the nutritional values of the oil. This research examines the oxidative stability of roasted and unroasted cold-pressed walnut oils under accelerated storage conditions. The oxidative stability of both oils was evaluated using physicochemical parameters: chemical composition (fatty acids, phytosterols, and tocopherols), pigment content (chlorophyll and carotenoids), specific extinction coefficients (K_232_ and K_270_), and quality indicators (acid and peroxide value) as well as the evaluation of radical scavenging activity by the DPPH method. The changes in these parameters were evaluated within 60 days at 60 ± 2 °C. The results showed that the levels of total phytosterols, the parameters of the acid and peroxide value, K_232_ and K_270_, increased slightly for both oils as well as the total tocopherol content and the antioxidant activity affected by the roasting process. In contrast, the fatty acid profiles did not change considerably during the 60 days of our study. After two months of oil treatment at 60 °C, the studied oils still showed an excellent physicochemical profile, which allows us to conclude that these oils are stable and can withstand such conditions. This may be due to the considerable content of tocopherols (vitamin E), which acts as an antioxidant.

## 1. Introduction

The common walnut (*Juglans regia* L.), Juglandaceae family, known also as English or Persian walnut, is a source of many bioactive compounds in many plant parts including the nut (seeds or kernels), leaves, flowers, shell, root, or bark, used in food, pharmaceutical, and cosmetic industries [1].

*Juglans regia* walnut oil, alongside its important economic value [2], is considered one of the richest oils in vitamins and antioxidant compounds [3], giving it many advantages in resistance to oxidation [4]. Walnut seeds represent a solid source of nutritional compounds [5] with several biological properties including antioxidant activity [6,7,8,9,10,11,12], anti-inflammatory activity [13,14,15,16,17,18,19,20], antibacterial activity [21,22,23], neuroprotective capacity [14,24,25,26] as well as having a great potential in preventing and reducing various types of cancer [8,27,28,29,30,31,32,33].

Although most of the articles mentioning the beneficial effects of walnuts are in vivo and in vitro experiments, new clinical studies describe the positive effects of walnut kernel consumption. In particular, they report that the long-term consumption of walnuts at ~15% of energy in the diet can reduce the concentrations of several inflammatory and metabolic syndrome biomarkers [34,35]; delay cognitive decline [36]; delay leukocyte telomere wear [37]; suppress breast cancer growth and survival [38]; reduce total mortality and CVD risk as well as increase life expectancy [39].

However, despite all these advantages, walnut oil, like all vegetable oils, can be unstable due to the sensitivity of its unsaturated fatty acids to oxidation. This oxidative sensitivity is regarded as one of the crucial parameters that indicate the quality, palatability, nutritional value, and toxicity of edible oils [40,41].

Several factors are cited as precursors of oil oxidation such as storage temperature, UV radiation, and oxygen in the air, which facilitate the oxidation of the unsaturated fatty acids by degrading their C–C double bonds, causing their transformation into secondary oxidation products such as ketones, aldehydes, and alcohols [40], and reducing their nutritional value [41]. On the other hand, roasting is one of the main pretreatments applied to oil seeds [42]. Different studies have shown the impact of roasting pretreatment on the composition of bioactive compounds such as phenols, sterols, and tocopherols as well as on the antioxidant activity and oxidative stability of oils [43,44,45,46,47].

This paper aimed to study the influence of both storage time at 60 °C (which can be considered as a stress condition and not a frying temperature and also the most used in various scientific papers with the same objective [48,49,50,51]) and traditional roasting on the physicochemical parameters, pigment content, and chemical composition as well as the antioxidant activity, in order to gain a better understanding of the stability behavior of walnut oils.

## 2. Materials and Methods

### 2.1. Plant Materials

The seeds of the *Juglans regia* plant studied in this paper were those from the Beni Mellal-Khenifra region—Morocco and, more precisely from the Azilal Province (31°58′00.0″ N, 6°34′00.0″ W), which is labelled as a geographical indication as “Azilal walnut”. After the fruits were harvested (September 2021), they were dried in the dark in a dry and ventilated area, peeled and then shelled manually to obtain only the kernels that were divided into two portions, one of which was treated with a traditional roasting process before the pressing stage and the other was directly cold-pressed. The extraction of oils was performed with the help of a cold press machine, Komet DD 85 G presses (IBG Monforts Oekotec GmbH, Mönchengladbach, Germany) to obtain two types of walnut oil: roasted walnut oil (RWO) and unroasted walnut oil (UWO). Samples of the collected oils were immediately analyzed, and the remainder were distributed in 30 mL non-transparent brown glass bottles well-filled to avoid the presence of air in contact with the oils. The bottles were placed in an oven (VWR, Sheldon manufacturing, INC. Cornelius, OR, USA) at a temperature of 60 ± 2 °C, and the analysis was repeated every 10 days in triplicate for two months.

### 2.2. Physicochemical Quality Parameters

The acid value (AV) was determined according to ISO 660 [52], the peroxide value was calculated in conformity with the ISO 3960 standard [53], while the specific extinction coefficient values (K_232_ and K_270_), were determined according to the American Oil Chemists’ Society (AOCS) recommended practice Ch 5-91 [54]. The acid value (AV) of an oil reflects the amount of potassium in mg required to neutralize its free acidity. The free acid content of fats increases with time, and this index, therefore, makes it possible to judge their state of deterioration; this was measured in mg of KOH/g of oil. Coefficients of specific extinction at 232 and 270 nm were measured using a 1% (*w*/*v*) solution of the oil in cyclohexane in 1 cm cell way length, utilizing a UV spectrophotometer (Model UV-5800PC, manufactured by Shanghai Metash Instruments Co., Ltd., Shanghai, China).

### 2.3. Chlorophylls and Carotenoids Content

Chlorophyll and carotenoid determinations were performed by preparing a 1% solution of walnut seed oil in cyclohexane, followed by measuring absorbance at 670 nm and 470 nm, respectively [55]. The contents were calculated using the following formulas (Equations (1) and (2)).

(1) $$\mathrm{Chlorophyll}\;\left(\mathrm{mg}/\mathrm{kg}\right)=\frac{\;A_{670}\times 10^{6}}{613\times 100\times d}$$

(2) $$\mathrm{Carotenoid}\;\left(\mathrm{mg}/\mathrm{kg}\right)=\frac{\;A_{470}\times 10^{6}}{2000\times 100\times d}$$

The chlorophyll and carotenoid contents were reported in ppm (mg of predominant pheophytin per kilogram of oil) and mg of lutein per kilogram of oil, respectively.

### 2.4. Fatty Acid Composition

Saturated, monounsaturated, and polyunsaturated fatty acids were determined following the recommended practices required by the American Oil Chemists’ Society (AOCS), Ce 1i-07 [54]. The fatty acids (FAs) undergo a transesterification reaction with KOH/methanol to convert into fatty acid methyl esters (FAMEs), which were later analyzed by gas chromatography with a capillary column (Varian CP-3800, Varian Inc., Middelburg, The Netherlands). The equipment had a polar stationary phase and split inlet system, the separation was according to the fatty acid chain length (CL), degree of unsaturation, and position of double bonds (which means the position isomers). A fused silica column was used with the following dimensions (l = 30 m, Ø = 0.32 mm). The carrier gas used was helium for chromatography, and the initial and final column temperatures were 170 °C and 230 °C, respectively, with a raising rate of 3 °C/min. The injected volume was 1 μL, and the detection was with a FID detector at 230 °C. The results were reported as percentages of each individual fatty acid present in the sample.

### 2.5. Tocopherol Composition

The amounts of tocopherol were quantified following the ISO 9936 standard method using an HPLC equipped with a fluorometric detector on a silica column (25 cm × 4 mm). An isooctane:isopropanol (99%:1%) mixture was used as the eluent with a debit of 1.2 mL/min for 20 min. Furthermore, the quantification was carried out with the help of standard external curves of α-, β-, γ-, and δ-tocopherols, and a quantitative and qualitative daily reference of tocopherol standards [56].

### 2.6. Phytosterols Composition

The sterol composition quantification was performed according to the Official AOCS Method Ch 6-91 [54], which requires saponification of each oil sample, then the extraction of the unsaponifiable fraction, followed by separation of the crude sterols by TLC, after the trimethylsilylation of the crude sterol fraction, and the determination of the sterol composition was performed by gas chromatography with a capillary column (Varian CP-3800, Varian Inc., Middelburg, The Netherlands). The column dimensions were l = 30 m, Ø = 0.32 mm)using helium as the carrier gas with an injection volume of 1 μL for each analysis.

### 2.7. Radical Scavenging Activity

The radical scavenging activity of RWO and UWO was evaluated against the DPPH (2,2-diphenyl 1-picrylhydrazyl) free radical using a slightly modified Gharibzahedi method [57]. Concisely, 100 mg of each oil (RWO and UWO) in 1 mL of toluene was vortexed for 30 s with 3900 µL of the DPPH toluene solution (10^−4^ M). The absorbance at 517 nm was measured after 30 min of mixing using a UV–Visible spectrophotometer (Model UV-5800PC UV/VIS Spectrophotometer, manufactured by Shanghai Metash Instruments Co. Ltd.). A mixture of 3900 µL DPPH solution and 1100 µL pure toluene was used as a negative control and the percentage of radical scavenging activity (%RSA) was calculated by Equation (3):

(3) $$\%\mathrm{RSA}=\frac{{A\;}_{\mathrm{control}\;}-{\;A}_{\;\mathrm{sample}}}{A_{\mathrm{control}}}\times 100$$

where A _Control_ is the absorbance of the negative control solution, and A _Sample_ is the absorbance of the sample solutions.

### 2.8. Statistical Analysis

GraphPad Prism 9 software was used to carry out the two-way ANOVA test for the verification of the statistical significance by Bonferroni’s multiple comparisons test at a confidence level of 95.0%, and the results were expressed as the means ± standard error of the mean of triplicate readings (*n* = 3). Differences at *p* > 0.05 were not considered statistically significant.

## 3. Results and Discussion

### 3.1. Physicochemical Quality Parameters, Chlorophylls, and Carotenoids Content

**Figure 1:**
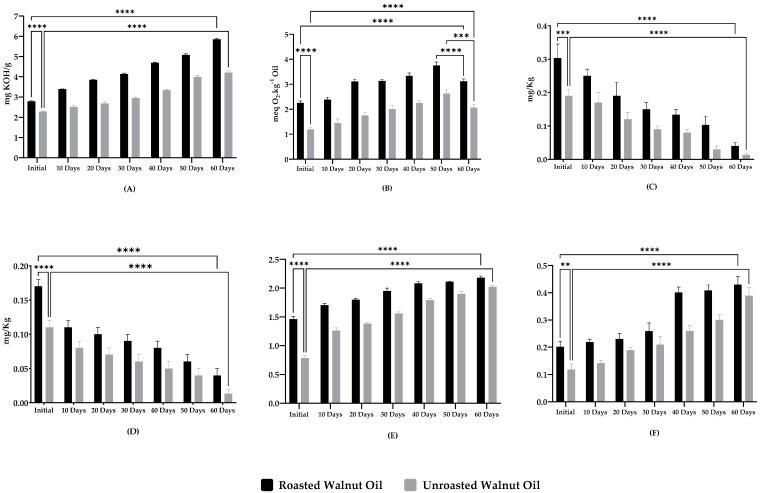
The physicochemical quality parameters, chlorophyll, and carotenoid content. (**A**) Evolution of acid value (mg KOH/g oil) during 60 days; (**B**) Evolution of peroxide value (meq O_2_·kg^-1^ oil) during 60 days; (**C**) Evolution of chlorophyll content (mg/kg) during 60 days; (**D**) Evolution of carotenoids content (mg/kg) during 60 days; (**E**) Evolution of K_232_ during 60 days; (**F**) Evolution of K_270_ during 60 days. ** (0.001 < *p* < 0.01); *** (0.0001 < *p* < 0.001); **** (*p* < 0.0001).

In Figure 1, we summarized the evolution of the contents of acid value, peroxide value, specific extinction coefficients (K_232_ and K_270_), chlorophyll, and carotenoids present in the oils (RWO and UWO) for 60 days. We started with the acid value (AV) results, which illustrated the quality and edibility of the oils [58]. It clearly appears that on the first day, the roasting process increased the acid value in a significant way with values of 2.78 ± 0.04 and 2.27 ± 0.02 for RWO and UWO, respectively (*p* < 0.0001), and at the end of the storage period. These values increased for both the RWO and UWO with an AV of 5.84 ± 0.04 mg KOH/g of oil and 4.20 ± 0.10 mg KOH/g of oil, respectively (Figure 1A), which were significantly different (*p* < 0.05). It is interesting to note that even under these storage conditions, the acid value of RWO did not exceed 4.00 mg KOH/g of oil, which is the acceptable limit of the Codex Alimentarius [59], until 30 days, while UWO could withstand more than 50 days before losing its conformity. It can be said that the roasting process facilitates the elevation of the acid value over time, while roasted and unroasted walnut oils are more stable in terms of acid value than bitter and sweet almond oils, which showed higher values than ours under the same storage conditions over a shorter period [48].

Concerning the peroxide value (PV), it represents one of the most widely used quality parameters to track the lipid oxidation and control the quality of the oil [60], providing a quantitative measure of the primary oxidation product levels in oils and fats [61]. According to the IOC (International Olive Council), 15 meq O_2_·kg^−1^ is the upper limit of acceptable oil quality [62]. Regarding the starting peroxide values, the UWO reached a value of 1.19 ± 0.09 meq O_2_·kg^−1^ while the RWO recorded a value of 2.25 ± 0.08 meq O_2_·kg^−1^, (Figure 1B) which means that the roasting process directly influences this parameter by increasing its value significantly (*p* < 0.0001). These values continued to increase until the 50^th^ day to find the highest value with 2.62 ± 0.15 meq O_2_·kg^−1^ and 3.75 ± 0,14 meq O_2_·kg^−1^ for the UWO and RWO, respectively, and after that. they dropped to 2.06 ± 0.13 meq O_2_·kg^−1^ for UWO and 3.12 ± 0.09 meq O_2_·kg^−1^ for RWO (*p* < 0.0001). This decrease (between the 50 and 60 days for both RWO and UWO) can be explained by the fact that the peroxide index provides quantitative measurements on the primary oxidation products. It can be observed that the values increased from the first day until the 50^th^, which is the peak value of this index (PV). After that, the products of primary oxidation underwent a second oxidation to transform to volatile secondary oxidation products such as aldehydes [63], which are not detectable by this parameter (PV). For this particular reason, a decrease in the values in a significant way (*p* < 0.0001) was observed. Overall, the findings revealed that roasting and storage time influenced the PV results by increasing its values, but at the end of the study, RWO and UWO still showed conforming values according to the International Olive Council below the limit of 15 meq O_2_·kg^−1^.

Regarding the pigment dosage, it is clear that roasting favors the transfer of pigments to the oil (Figure 1C,D), and this is in line with the conclusions found by Adrián in 2018 [64]. The analysis by UV–Visible spectrophotometer detects higher pigment contents in RWO than in UWO (*p* < 0.05). Beginning with the determination of chlorophyll, which is a coloring substance that plays an important role in the antioxidant activity of vegetable oils [65]. The values recorded at the beginning of the test were 0.30 mg/kg and 0.19 mg/kg for the RWO and UWO, respectively (*p* < 0.001). However, these concentrations decreased progressively with time and dropped at the end of storage by more than 86% for the RWO and 94% of the initial value for the UWO (*p* < 0.0001), and this lowering of values may be sufficient to induce a slight rise in the oxidation rate of the oils [66]. Carotenoid molecules are also potent antioxidants [67], since they can scavenge singlet oxygen and provide various health benefits that include reducing the risk of chronic diseases such as degenerative eye and cardiovascular diseases [68,69,70]. Since carotenoids are highly unsaturated, they can degrade rapidly when stored under stressful conditions [71]. In this paper, carotenoids followed the same trend as chlorophyll, where RWO and UWO showed a loss at the end of the study of more than 76% and 90%, respectively (*p* < 0.0001), and these losses were justified by the high storage temperature associated with prolonged storage time, in line with what Agyare found in 2022 [72].

Figure 1E,F illustrate the absorption values at 232 nm (K_232_) and 270 nm (K_270_), respectively, which can be considered as an image of the oxidation state of vegetable oils, indicating the evolution of the formation of primary and secondary oxidation products [73]. A higher extinction value of 232 nm means that the product is more peroxidized. Likewise, the higher the extinction value at 270 nm, the richer in secondary oxidation products, and the worse its conservation properties [55]. It can be seen that roasting and storage time in the applied conditions directly impact the increase in the values of K_232_ and K_270_, which signifies an increase in the oxidation level (*p* < 0.05) and a decrease in the quality of these oils. Starting with K_232_ and after 60 days of storage at 60 °C, the values increased from 1.46 ± 0.04 to 2.18 ± 0.03 for RWO and from 0.78 ± 0.04 to 2.03 ± 0.03 for UWO, which were significantly different (*p* < 0.05). This indicates an increase in the amount of primary oxidation product, but despite this increase, it remains within the acceptable limit for extra virgin olive oil, below the 2.5 required by the IOC [62,74]. On the other hand, K_270_ follows the same evolution as K_232_ with a duplication of the value from 0.20 ± 0.02 to 0.43 ± 0.03 for RWO (*p* < 0.0001), and a threefold increase for UWO from 0.11 ± 0.02 to 0.38 ± 0.03 (*p* < 0.0001). We noted that our results exceeded the value of 0.25 at the end of our investigation, which is the acceptable limit of extra virgin olive oil asked by the IOC [62]. This increase leads to roasted and unroasted walnut oils losing their conformity due to the transformation of the peroxides formed previously to volatile products such as ketones and aldehydes, which are known as secondary oxidation products [75,76].

### 3.2. Fatty Acid Composition

*Juglans regia* oil is considered as one of the most important oils in the human diet because of its essential fatty acid composition [77,78], specifically the mono- and polyunsaturated fatty acids that have several medicinal effects including the regulation of blood lipids and thrombus cleansing [79]. Changes in the fatty acid composition of roasted and unroasted walnut oils during 60 days of storage at 60 °C are reported in Table 1. In this study, the five main fatty acids in *Juglans regia* oil were compared: palmitic acid (C16:0), stearic acid (C18:0), oleic acid (C18:1), linoleic acid (C18:2), and linolenic acid (C18:3). Our findings showed a high percentage of polyunsaturated fatty acids (C18:2 and C18:3) exceeding 70%, 15% of monounsaturated fatty acids, and 13% of saturated fatty acids. The main unsaturated fatty acid in the studied oils was linoleic acid (C18:2) with percentages between 58.86 ± 0.04% and 60.99 ± 0.03% in the UWO (*p* < 0.05) and between 60.22 ± 0.02% and 60.43 ± 0.02% in the RWO (*p* < 0.05). In terms of saturated fatty acids, the major one is palmitic acid (C16:0) between 8.40 ± 0.02% and 9.44 ± 0.02% in UWO (*p* < 0.05) and between 8.57 ± 0.03% and 9.90 ± 0.03% in RWO (*p* < 0.05). Therefore, it can be said that statistically, the interaction between the roasting effect and storage time in the applied conditions affects the fatty acid composition. However, this impact remains minor, considering that C18:2 is consistently the major fatty acid followed by C18:1, C18:3, C16:0, and C18:3 in accordance with the results found in different walnut cultivars [80,81] and in both the RWO and UWO oils during the whole period of this investigation.

### 3.3. Tocopherol Composition

In recent years, antioxidant molecules have attracted considerable interest and among these, molecules stand out the tocopherols, which are known as one of the most essential antioxidants in nutrition due to their liposolubility [82] and their various biological activities [83]. These include the prevention of various types of cancer [84,85,86,87,88,89,90] and cardiovascular disease [91,92,93], and all these benefits have led us to be interested in the composition and evolution of these tocopherol molecules and all their isomers. Table 2 shows the contents of α, γ, and δ-tocopherol as well as the total tocopherol values in mg/kg. It is noteworthy that β-tocopherol was not detected in the *Juglans regia* oils or is present in trace amounts, in accordance with what was found by Cecilia Cittadini in 2020 for Argentinian walnut oil [94]. γ-Tocopherol was detected as the primary tocopherol in both RWO and UWO, followed by δ and α-tocopherol, respectively. In terms of the values of γ-tocopherol, it ranged between 606.32 ± 1.80 mg/kg and 508.68 ± 3.20 mg/kg in UWO (*p* < 0.05) and between 624.77 ± 2.30 mg/kg and 579.56 ± 2.20 mg/kg in RWO (*p* < 0.05). These results are higher than those recorded by Bou Abdallah for six different Tunisian walnut cultivars [95]. Regarding the contents of total tocopherols, our results showed that at the beginning and without the effects of storage time, the contents of the total tocopherols increased from 669.45 ± 2.50 mg/kg in UWO to 680.50 ± 2.05 mg/kg in RWO (*p* < 0.05). Similarly, at the end of this study, we recorded values of 639.80 ± 1.45 mg/kg in UWO and 665.24 ± 1.10 mg/kg in RWO (*p* < 0.05). This indicated that roasting had a positive effect on increasing the total tocopherol content, which can be explained by the fact that during roasting, there is damage at the membrane, which leads to the release of a more significant amount of these molecules (tocopherols) in oil [96,97]. This subsequently explains the increase in the antioxidant activity of roasted seed oils compared to the unroasted ones. According to the effect of the storage time at 60 °C, the results showed that the oil retained almost the same content of total tocopherols with a loss of 4.43% for UWO and 2.24% for RWO, which means that the studied oils are stable and can resist in such storage conditions.

### 3.4. Sterol Composition

Walnut (*Juglans regia*) oil is a highly recommended oil due to its richness in phytosterol, which has the capacity to prevent cardiovascular diseases [48,98]. These molecules are more hydrophobic than cholesterol and can dislodge cholesterol from intestinal micelles and reduce LDL absorption. In combination with linolenic acid, phytosterols have both complementary and synergistic lipid-lowering effects [35], and also has an impressive antioxidant activity [99,100,101]. The individual and total sterol composition of UWO and RWO during the different storage times under the applied conditions are summarized in Table 3. The most predominant phytosterol is β-sitosterol, followed by Δ5-avenasterol and campesterol, confirmed by various compositional studies of walnut oil [102,103,104]. During storage at 60 °C, the amount of the significant phytosterols, β-sitosterol, decreased with losses of 13.78% in UWO, from 1058.09 mg/kg to 912.23 mg/kg and 17.49% in RWO, from 1056.98 mg/kg to 872.07 mg/kg. The same goes for the total sterol contents, which also dropped by 15.06% in UWO, from 1260.38 mg/kg to 1070.57 mg/kg, and by 17.42% in RWO from 1236.66 mg/kg to 1021.28 mg/kg. All of these results were significantly different at *p* < 0.05. Therefore, we can affirm that our storage conditions negatively impacted on the phytosterol content, similar to what was previously found in the sweet and bitter almond oil study [48]. Roasting also had a negative influence on individual and total sterols, similar to what was found previously in a study on sesame oil [105], which justifies this by the oxidation of these compounds at high roasting temperatures. This is in contrast to another study that found a higher level in roasted flaxseed oil compared to the unroasted ones. We can attribute these results to the cell structure damage created during the roasting process, which subsequently leads to an enhancement in the extractability of phytosterols [106]. However, it is important to note that even under these storage conditions (60 °C for 60 days) and the roasting process, walnut oils (UWO and RWO) maintained their phytosterol content with a slight decrease.

### 3.5. Antioxidant Activity

**Figure 2:**
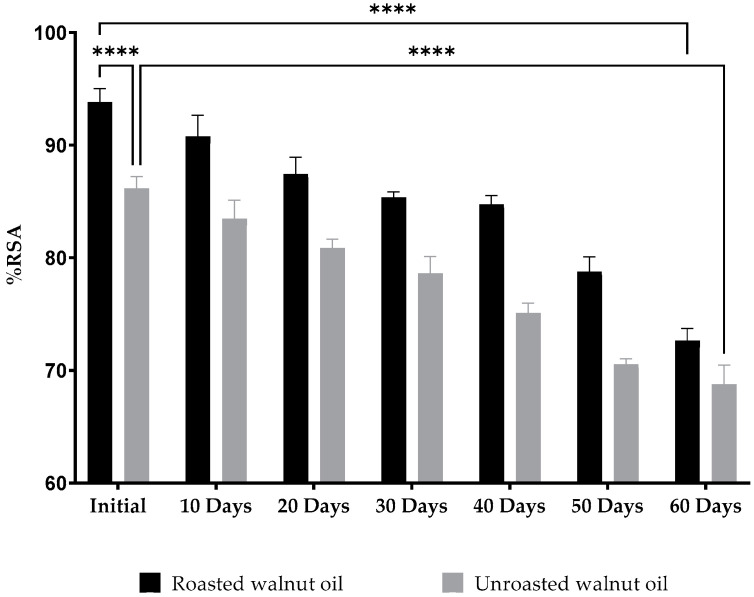
The evolution of the radical scavenging activity (RSA) during 60 days. **** (*p* < 0.0001).

In this study, changes in the spectrometric absorbance were used to plot the histogram of radical scavenging activity evolution (Figure 2). However, our results showed that walnut oil had a very high antioxidant capacity in accordance with the literature [107,108,109,110], with RSA percentages above 60% for all of the samples. Initial values of %RSA were equal to 86.15 ± 1.08% for UWO and 94.81 ± 1.20% for RWO, which were significantly different (*p* < 0.0001). Contrary to what we expected, it can be easily concluded that, initially, roasting has a direct impact on antioxidant activity by increasing the power and capacity of the oil to scavenge the DPPH free radicals. These results can be explained by the fact that during the roasting process, Maillard reaction products (MRPs) are formed after the initiation of chemical reactions between reducing sugars and amino acids present in the oils [111,112], which leads to a significant enhancement in the antioxidant capacity [113,114]. However, this activity will decrease with time, under the influence of the temperature applied during the storage period (two months) with a loss of 17% for RWO and 13% for UWO at the end of the study (*p* < 0.0001). This may be due to the considerable content of tocopherols (vitamin E) in *Juglans regia* oil [115,116,117], which acts as an essential antioxidant that protects the oils from degrading and losing their properties [118].

## 4. Conclusions

The effects of roasting and accelerated storage conditions were investigated in this study. Acid value, peroxide value, and specific extinction (K_232_ and K_270_) increased with the roasting and storage time. Roasting enhanced the pigment transfer into the oil, which increased the chlorophyll and carotenoid values, while these values decreased with storage time. The total sterol content slightly reduced, influenced by both factors applied in this study. While the total content of tocopherols was increased by the roasting process, this increased content of tocopherols was strongly correlated with the antioxidant activity, which leads to an increase in the percentage of the radical scavenging activity (%RSA) of walnut oil against the DPPH radical. However, all of the parameters evaluated in this article were negatively affected by the storage conditions applied. At the same time, the roasting process has shown an important role in increasing the vitamin E content and the antioxidant capacity, preventing oxidation and degradation of the investigated oils, and increasing its nutritional value. In spite of all the valuable information provided in this article, further research should be considered as perspectives to further valorize these oils, among these, a study on the stability of walnut seed oil under the influence of different roasting temperatures as well as the effect of UV radiation on the photodegradation of bioactive compounds in walnut seeds oils, which are currently in progress. However, other studies are still needed to valorize Moroccan walnut oils in the pharmacological field by evaluating the antioxidant activity against a wide range of free radicals (DPPH, ABTS, FRAP, ORAC, etc.) as well as the antimicrobic, antifungal, and antidiabetic activities of these oils. This research will improve the understanding of the stability behavior of cold-pressed walnut oils, which will facilitate their preservation and storage as well as their applications in various nutritional and pharmacological fields.

## Figures and Tables

**Table 1:** The fatty acid composition (%) of UWO and RWO during 60 days of storage at 60 ± 2 °C.

	C16:0	C18:0	C18:1	C18:2	C18:3	SFA/UFA
***Unroasted Walnut (Juglans regia *L.*) Seed Oils***						
**Initial**	9.90 ± 0.03 ^1,a^	2.67 ± 0.05 ^1,a^	14.65 ± 0.03 ^1,a^	58.86 ± 0.04 ^1,a^	13.42 ± 0.01 ^1,a^	0.1
**10**	8.58 ± 0.01 ^1,b^	2.41 ± 0.02 ^1,b^	14.83 ± 0.02 ^1,b^	60.23 ± 0.02 ^1,b^	13.28 ± 0.02 ^1,b^	0.1
**20**	8.61 ± 0.02 ^1,b^	2.37 ± 0.02 ^1,b^	14.61 ± 0.02 ^1,a^	60.28 ± 0.01 ^1,b,c^	13.52 ± 0.02 ^1,c^	0.1
**30**	8.43 ± 0.03 ^1,c^	2.34 ± 0.02 ^1,b^	14.67 ± 0.03 ^1,a^	60.48 ± 0.03 ^1,d^	13.52 ± 0.01 ^1,c^	0.1
**40**	8.23 ± 0.02 ^1,d^	2.39 ± 0.01 ^1,b^	14.77 ± 0.03 ^1,b^	60.35 ± 0.04 ^1,c^	13.66 ± 0.02 ^1,d^	0.1
**50**	8.32 ± 0.03 ^1,e^	2.38 ± 0.01 ^1,b^	14.83 ± 0.02 ^1,b^	60.50 ± 0.02 ^1,d^	13.58 ± 0.03 ^1,c,d^	0.1
**60**	8.57 ± 0.02 ^1,b^	2.41 ± 0.02 ^1,b^	15.01 ± 0.03 ^1,c^	60.99 ± 0.03 ^1,e^	12.54 ± 0.02 ^1,e^	0.1
***Roasted Walnut (Juglans regia *L.*) Seed Oils***						
**Initial**	9.44 ± 0.02 ^2,A^	2.32 ± 0.03 ^2,A^	14.71 ± 0.03 ^1,A^	60.22 ± 0.02 ^2,A^	12.70 ± 0.04 ^2,A^	0.1
**10**	8.64 ± 0.02 ^1,B^	2.37 ± 0.02 ^1,A,B^	14.50 ± 0.03 ^2,B^	60.44 ± 0.01 ^2,B^	13.39 ± 0.05 ^2,B^	0.1
**20**	8.79 ± 0.03 ^2,C^	2.31 ± 0.01 ^1,A^	14.90 ± 0.03 ^2,C^	60.47 ± 0.02 ^2,B^	12.91 ± 0.03 ^2,C^	0.1
**30**	8.94 ± 0.02 ^2,D^	2.36 ± 0.02 ^1,A,B^	15.21 ± 0.02 ^2,D^	59.55 ± 0.02 ^2,C^	13.37 ± 0.02 ^2,B^	0.1
**40**	8.52 ± 0.01 ^2,E^	2.40 ± 0.01 ^1,B^	14.55 ± 0.01 ^2,B,E^	60.32 ± 0.01 ^1,D^	13.60 ± 0.02 ^1,D^	0.1
**50**	8.49 ± 0.01 ^2,E^	2.33 ± 0.02 ^1,A,B,C^	14.60 ± 0.02 ^2,E^	60.49 ± 0.01 ^1,B^	13.55 ± 0.03 ^1,D,E^	0.1
**60**	8.40 ± 0.02 ^2,F^	2.41 ± 0.03 ^1,B^	14.58 ± 0.01 ^2,E^	60.43 ± 0.02 ^1,B^	13.51 ± 0.01 ^2,E^	0.1

Data with the same superscript numbers in the same column for roasting in the same period were not significantly different (*p* > 0.05). Data with the same lowercase letters in the same column for time of storage of UWO were not significantly different (*p* > 0.05). Data with the same uppercase letters in the same column for time of storage of RWO were not significantly different (*p* > 0.05).

**Table 2:** The tocopherol composition (mg/kg) of UWO and RWO during 60 days of storage at 60 ± 2 °C.

	α-Tocopherol	γ-Tocopherol	δ-Tocopherol	Total
***Unroasted Walnut (Juglans regia *L.*) Seed Oils***				
**Initial**	9.37 ± 0.08 ^1,a^	606.32 ± 1.80 ^1,a^	50.74 ± 0,93 ^1,a^	669.45 ± 2.50 ^1,a^
**10**	7.25 ± 0.10 ^1,b^	609.02 ± 2.40 ^1,a^	47.28 ± 1.65 ^1,a^	665.01 ± 3.33 ^1,a,b^
**20**	9.47 ± 0.06 ^1,a,d^	604.54 ± 1.90 ^1,a^	48.57 ± 1.54 ^1,a^	662.58 ± 2.13 ^1,b^
**30**	10.97 ± 0.13 ^1,c^	605.28 ± 1.60 ^1,a^	44.61 ± 0.90 ^1,b^	660.86 ± 1.96 ^1,b,c^
**40**	9.76 ± 0.05 ^1,d^	597.71 ± 0.90 ^1,b^	44.95 ± 1.05 ^1,b^	655.24 ± 2.85 ^1,c^
**50**	9.03 ± 0.05 ^1,e^	584.93 ± 2.80 ^1,c^	48.77 ± 0.85 ^1,a,c^	645.12 ± 3.09 ^1,d^
**60**	8.88 ± 0.11 ^1,e^	580.68 ± 3.20 ^1,c^	49.91 ± 1.30 ^1,c^	639.80 ± 1.45 ^1,d^
				**Loss (%) = 4.43**
***Roasted Walnut (Juglans regia *L.*) Seed Oils***				
**Initial**	9.60 ± 0.20 ^1,A^	624.77 ± 2.30 ^2,A^	46.07 ± 0.75 ^2,A^	680.50 ± 2.05 ^2,A^
**10**	8.19 ± 0.13 ^2,B^	621.70 ± 1.90 ^2,A^	46.83 ± 1.20 ^1,A^	676.72 ± 1.60 ^2,A,B^
**20**	7.47 ± 0.05 ^2,C^	619.66 ± 0.93 ^2,A^	45.68 ± 1.05 ^1,A,B^	672.81 ± 0.95 ^2,B,C^
**30**	9.16 ± 0.08 ^2,D^	611.76 ± 1.50 ^2,B^	47.45 ± 0.80 ^1,A^	668.37 ± 0.88 ^2,C^
**40**	8.65 ± 0.11 ^2,E^	605.73 ± 2.50 ^1,B^	44.84 ± 1.44 ^1,A^	660.41 ± 0.53 ^1,D^
**50**	10.35 ± 0.12 ^2,F^	598.34 ± 1.70 ^2,C^	45.38 ± 0.43 ^1,A,B^	662.67 ± 0.90 ^2,C,D^
**60**	11.97 ± 0.09 ^2,G^	579.56 ± 2.20 ^1,D^	42.38 ± 0.94 ^2,B^	665.24 ± 1,10 ^2,C,D^
				**Loss (%) = 2.24**

Data with the same superscript numbers in the same column for roasting in the same period were not significantly different (*p* > 0.05). Data with the same lowercase letters in the same column for time of storage of UWO were not significantly different (*p* > 0.05). Data with the same uppercase letters in the same column for time of storage of RWO were not significantly different (*p* > 0.05).

**Table 3:** The sterol composition (mg/kg) of UWO and RWO during 60 days of storage at 60 ± 2 °C.

	Cholesterol	Campesterol	Stigmasterol	β-Sitosterol	Δ5-Avenasterol	Δ7-Stigmasterol	Δ7-Avenasterol	Total Sterols
***Unroasted Walnut (Juglans regia *L.*) Seed Oils***								
**Initial**	26.47 ± 0.03 ^1,a^	64.28 ± 0.15 ^1,a^	3.53 ± 0.001 ^1,a^	1058.09 ± 1.02 ^1,a^	70.58 ± 0.07 ^1,a^	0.63 ± 0.001 ^1,a^	1.51 ± 0.001 ^1,a^	1260.38 ± 1.22 ^1,a^
**10 Days**	5.13 ± 0.01 ^1,b^	62.75 ± 0.07 ^1,b^	4.64 ± 0.001 ^1,b^	1048.85 ± 1.13 ^1,b^	72.52 ± 0.08 ^1,b^	4.40 ± 0.001 ^1,b^	1.10 ± 0.001 ^1,b^	1220.87 ± 1.31 ^1,b^
**20 Days**	13.25 ± 0.02 ^1,c^	59.21 ± 0.07 ^1,c^	9.26 ± 0.01 ^1,c^	993.02 ± 1.19 ^1,c^	59.09 ± 0.07 ^1,c^	1.03 ± 0.001 ^1,c^	5.39 ± 0.01 ^1,c^	1172.4 ± 1.40 ^1,c^
**30 Days**	29.05 ± 0.04 ^1,d^	64.21 ± 0.09 ^1,a^	11.46 ± 0.02 ^1,d^	957.01 ± 1.40 ^1,d^	65.04 ± 0.09 ^1,d^	1.14 ± 0.001 ^1,d^	2.88 ± 0.001 ^1,d^	1150.51 ± 1.68 ^1,d^
**40 Days**	14.69 ± 0.01 ^1,e^	59.84 ± 0.05 ^1,d^	7.50 ± 0.01 ^1,e^	912.11 ± 0.81 ^1,e^	53.86 ± 0.05 ^1,e^	2.34 ± 0.001 ^1,e^	1.78 ± 0.001 ^1,e^	1072.39 ± 0.95 ^1,e^
**50 Days**	13.53 ± 0.01 ^1,f^	60.36 ± 0.06 ^1,e^	6.61 ± 0.01 ^1,f^	919.54 ± 0.90 ^1,f^	53.80 ± 0.05 ^1,e^	1.97 ± 0.001 ^1,f^	1.51 ± 0.001 ^1,a^	1070.09 ± 1.05 ^1,e^
**60 Days**	14.67 ± 0.02 ^1,e^	58.02 ± 0.07 ^1,f^	3.32 ± 0.01 ^1,g^	912.23 ± 1.14 ^1,e^	60.81 ± 0.07 ^1,f^	0.95 ± 0.001 ^1,g^	1.43 ± 0.001 ^1,f^	1070.57 ± 1.33 ^1,e^
***Roasted Walnut (Juglans regia *L.*) Seed Oils***								
**Initial**	6.06 ± 0.01 ^2,A^	59.98 ± 0.06 ^2,A^	4.82 ± 0.01 ^2,A^	1056.98 ± 1.10 ^1,A^	69.38 ± 0.07 ^2,A^	12.24 ± 0.01 ^2,A^	1.24 ± 0.001 ^2,A^	1236.66 ± 1.29 ^2,A^
**10 Days**	14.10 ± 0.01 ^2,B^	59.00 ± 0.06 ^2,B^	2.96 ± 0.001 ^2,B^	1013.16 ± 0.95 ^2,B^	55.44 ± 0.05 ^2,B^	2.13 ± 0.001 ^2,B^	0.24 ± 0.001 ^2,B^	1184.7 ± 1.11 ^2,B^
**20 Days**	10.09 ± 0.01 ^2,C^	56.56 ± 0.06 ^2,C^	2.99 ± 0.001 ^2,C^	977.70 ± 1.05 ^2,C^	62.89 ± 0.07 ^2,B^	0.97 ± 0.001 ^2,C^	1.25 ± 0.001 ^2,C^	1132.7 ± 1.21 ^2,C^
**30 Days**	16.68 ± 0.02 ^2,D^	54.44 ± 0.07 ^2,D^	4.28 ± 0.01 ^2,D^	937.00 ± 1.17 ^2,D^	62.88 ± 0.08 ^2,C^	0.10 ± 0.001 ^2,D^	0.91 ± 0.001 ^2,D^	1097.32 ± 1.37 ^2,D^
**40 Days**	9.56 ± 0.02 ^2,E^	53.80 ± 0.07 ^2,E^	2.94 ± 0.001 ^2,B^	902.25 ± 1.23 ^2,E^	60.94 ± 0.08 ^2,D^	0.95 ± 0.001 ^2,E^	1.37 ± 0.001 ^2,E^	1050.72 ± 1.43 ^2,E^
**50 Days**	14.17 ± 0.02 ^2,F^	58.58 ± 0.06 ^2,F^	2.26 ± 0.001 ^2,E^	891.68 ± 0.97 ^2,F^	59.59 ± 0.06 ^2,E^	0.83 ± 0.001 ^2,F^	1.27 ± 0.001 ^2,F^	1038.51 ± 1.13 ^2,F^
**60 Days**	15.52 ± 0.01 ^2,G^	50.66 ± 0.02 ^2,G^	3.98 ± 0.001 ^2,F^	872.07 ± 0.43 ^2,G^	58.52 ± 0.03 ^2,F^	0.09 ± 0.001 ^2,G^	0.84 ± 0.001 ^2,G^	1021.28 ± 0.50 ^2,G^

Data with the same superscript numbers in the same column for roasting in the same period were not significantly different (*p* > 0.05). Data with the same lowercase letters in the same column for time of storage of UWO were not significantly different (*p* > 0.05). Data with the same uppercase letters in the same column for time of storage of RWO were not significantly different (*p* > 0.05).

## Data Availability

All data were included in this manuscript.
